# Trends in Health Policy and Systems Research over the Past Decade: Still Too Little Capacity in Low-Income Countries

**DOI:** 10.1371/journal.pone.0027263

**Published:** 2011-11-22

**Authors:** Taghreed Adam, Saad Ahmad, Maryam Bigdeli, Abdul Ghaffar, John-Arne Røttingen

**Affiliations:** 1 Alliance for Health Policy and Systems Research, World Health Organization, Geneva, Switzerland; 2 Harvard Kennedy School, Cambridge, Massachusetts, United States of America; 3 Institute for Health and Society, University of Oslo, Oslo, Norway; Kenya Medical Research Institute - Wellcome Trust Research Programme, Kenya

## Abstract

**Background:**

The past decade has seen several high-level events and documents committing to strengthening the field of health policy and systems research (HPSR) as a critical input to strengthening health systems. Specifically, they called for increased production, capacity to undertake and funding for HPSR. The objective of this paper is to assess the extent to which progress has been achieved, an important feedback for stakeholders in this field.

**Methods and Finding:**

Two sources of data have been used. The first is a bibliometric analysis to assess growth in production of HPSR between 2003 and 2009. The six building blocks of the health system were used to define the scope of this search. The second is a survey of 96 research institutions undertaken in 2010 to assess the capacity and funding availability to undertake HPSR, compared with findings from the same survey undertaken in 2000 and 2008. Both analyses focus on HPSR relevant to low-income and middle-income countries (LMICs). Overall, we found an increasing trend of publications on HPSR in LMICs, although only 4% were led by authors from low-income countries (LICs). This is consistent with findings from the institutional survey, where despite improvements in infrastructure of research institutions, a minimal change has been seen in the level of experience of researchers within LIC institutions. Funding availability in LICs has increased notably to institutions in Sub-Saharan Africa; nonetheless, the overall increase has been modest in all regions.

**Conclusion:**

Although progress has been made in both the production and funding availability for HPSR, capacity to undertake the research locally has grown at a much slower pace, particularly in LICs where there is most need for this research. A firm commitment to dedicate a proportion of all future funding for research to building capacity may be the only solution to turn the tide.

## Introduction

**Figure 1:**
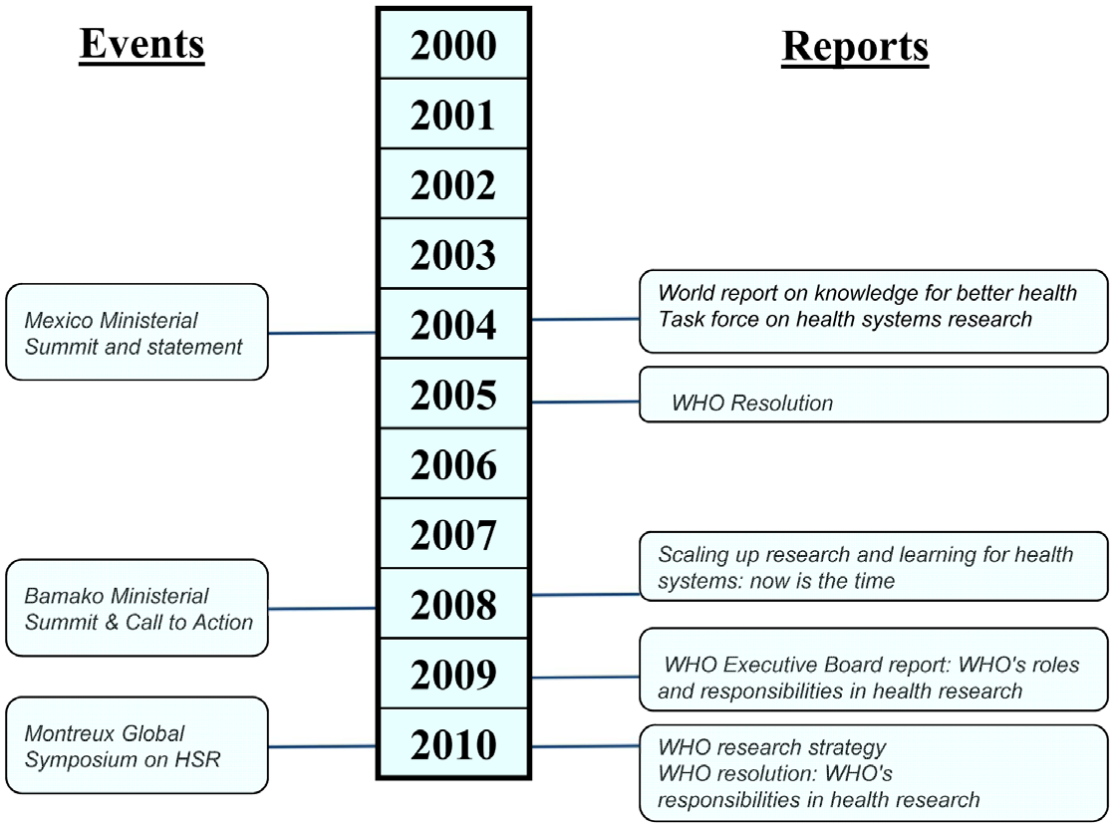
Timeline of major events or reports related to HPSR between 2000–2010.

The importance of invigorating the field of health policy and systems research (HPSR) has been increasingly emphasized in several action-oriented reports and events over the past decade. Notable examples include the 2004 Ministerial Summit for Health Research in Mexico and its proceedings [1]–[3], subsequent WHO strategic reports and resolutions [4], [5], the 2008 Global Ministerial Forum on Research for Health in Bamako and its proceedings [6]–[9], the 2010 WHO research strategy [10], [11], recommendations for future funding strategies of major funding entities [12]–[14], and most recently, the 2010 First Global Symposium on Health Systems Research (HSR) [15], [16], see Figure 1.

More specifically, all of these reports and events consistently called for: 1) Increased funding for HSR; 2) Increased institutional capacity for HSR; and 3) Knowledge production in HSR [4], [6], [7], [9], [15]. Health policy research has been explicitly included and linked to health systems research in more recent documents [10], [12], [13], [17].

By HPSR we refer to the production of new knowledge to improve how societies organize themselves in achieving collective health goals, and how different actors interact in the policy and implementation processes to contribute to policy outcomes [18], [19]. HPSR is characterized by the types of questions it addresses rather than any particular methodologies. It focuses primarily upon policies, organizations and programmes but not the clinical management of patients or basic biomedical research. HPSR can address any or several of the health systems building blocks and their ultimate objective to promote the coverage, quality, efficiency and equity of health systems [20].

The objective of this paper is to evaluate how the field of HPSR, particularly on issues relevant to and produced in low and middle income countries (LMICs), has evolved over the past decade with respect to the three dimensions listed above. The findings will inform global and national stakeholders about progress towards achieving their aspirations and commitments. It will also inform the upcoming 2012 World Health Report and the first Global Strategy on Health Systems Research to be published in 2012 with the achievements and gaps in this important area of research.

## Methods

We assessed the progress in the field of HPSR with respect to three dimensions: 1) Knowledge production in HPSR; 2) Institutional capacity for HPSR; and 3) Funding for HPSR. Two sources of data were used. The first is a bibliometric analysis to assess the growth of publications on HPSR relevant to LMICs during the past decade. This addresses dimension one above. The second source is a survey of research institutions involved in HPSR relevant to LMICs, to assess the capacity and funding availability for HPSR. The survey addresses dimensions two and three above.

### Bibliometric analysis

A bibliometric analysis was conducted in PubMed to retrieve publications relevant to HPSR in LMICs. The six building blocks of the health system as defined by the World Health Organization were used to define the scope of this search. These are: human resources, health financing, service delivery, health information systems, medicines and technologies and governance [20]. Publications between 2003 and 2009 were retrieved– 2003 being one year before the most important reports and events emphasizing the importance of re-invigorating the field of HPSR (Figure 1), and 2009, the latest year for which indexed publications from PubMed could be obtained at the time of this analysis. Only publications focusing on LMIC contexts were included. No language restriction was set.

A detailed and comprehensive list of search terms was developed, building on and updating available search strategies addressing the six building blocks of the health system [21]–[23]. Details of the search terms and the search strategy are available in Annex S1 (Search strategy). The main topic areas searched under each building block is summarized in Table 1. All building blocks were fairly represented except for medicines and technologies, where we focused our search on topics related to medicines.

**Table 1:** Topics explored in the bibliometric analysis by health systems building block.

Topics	
**Human resources**	**Medicines**
– Distribution and retention	– Monitoring (e.g., adverse reactions)
– Training (pre-service and in-service)	– Selection (e.g., in essential drug lists)
– Migration	– Regulation and Quality Assurance
**Health financing**	– Intellectual Property
– Payment mechanisms	– Access
– Health insurance	– Policy/Reform (e.g., national drug policies)
– Resource allocation	– Insurance and Financing
**Governance and leadership**	– Medicine Supply (e.g., forecasting)
– Government regulation and legislation	– Prescribing and Utilization
– Licensing and accreditation	– Information (e.g., for education and advocacy)
– Professional authority and roles (e.g., scope, content and location of practice)	– Marketing(e.g., drug promotion)
– Audit	**Service delivery**
– Consumer involvement	– Access, integrated care, continuum of care and modes of delivery
**Information systems**	– Non-state sector (e.g., contracting, private sector)
– Medical and drug records; Computerized records; and management information systems	– Quality of care and performance

The retrieved articles were downloaded from PubMed as text files and were converted to a database in Excel using reference manager software [24]. Data were cleaned and analysed in Excel. Retrieved articles were categorized by publication year, country of residence of corresponding author and publication topic. Information on residence of corresponding author was further categorized by income group using the World Bank classification. Trends in volume and nature of publications were analysed with respect to these categories.

### Institutional survey

In September 2010, an invitation email was sent to 279 contact persons in research institutions involved in HPSR in LMICs, introducing the objectives of the survey and providing access to it. The mailing list included contact persons in partner institutions of the Alliance for HPSR [19], grantees funded by the Alliance if their institutions are not already included in the first list, and other institutions active in the field of HPSR identified to us through our contacts in the previous two lists. Criteria for inclusion was limited to institutions undertaking HPSR relevant to LMICs regardless of where the institution is based, i.e., institutions in high-income countries (HICs) were also included. Four email reminders were sent to encourage participants to respond to the survey. 112 responses were received (40% response rate) of which 96 were valid.

The survey included sections on human resource availability by type of discipline and professional degree; availability of infrastructure conducive to higher quality research such as availability of computers, internet and access to peer-reviewed publications; total number of publications and presentations on HPSR in the previous year; total number of grants related to HPSR in LMICs in the previous year and details on the latest 5 grants; and finally their perception of the availability of funds for and interest in undertaking HPSR. The survey data were imported, checked, and cleaned using Microsoft Excel and Stata 9 software [25]. Outliers were checked for accuracy and respondents were contacted to confirm any queries. Data was analysed using Stata software.

A similar group of research institutions was previously surveyed in 1999–2001 and 2008 using the same questionnaire, providing a useful metric to compare findings from the 2010 survey [9], [26]. Since 76% of the data from the first survey was from 2000, we consider it the base year for comparison purposes. Institutions based in HICs were only included in the 2008 and 2010 surveys while the 2000 survey was only limited to those based in low-income (LICs) or middle-income countries (MICs). There was minimal overlap (8% and 27% of institutions included in 2010 also responded to the 2000 and 2008 surveys respectively) between institutions included in the three surveys. This can be explained by the expanding list of Alliance partner institutions over the years, where newer partners are more likely to respond than older ones; the generally low response rate; and loss of contact with some institutions due to staff turnover. However, a balanced regional representation was obtained in the three surveys.

The survey data were categorized by income level using the World Bank income classification. To account for inflation, current US dollars in 2000 and 2008 were converted to 2010 prices using GDP deflators [27].

Ethics approval was not sought for this study since the data collected during the institutional survey represented basic information on staffing and funding availability and did not represent any risks to the participants or their institutions.

## Results

### Bibliometric analysis

**Figure 2:**
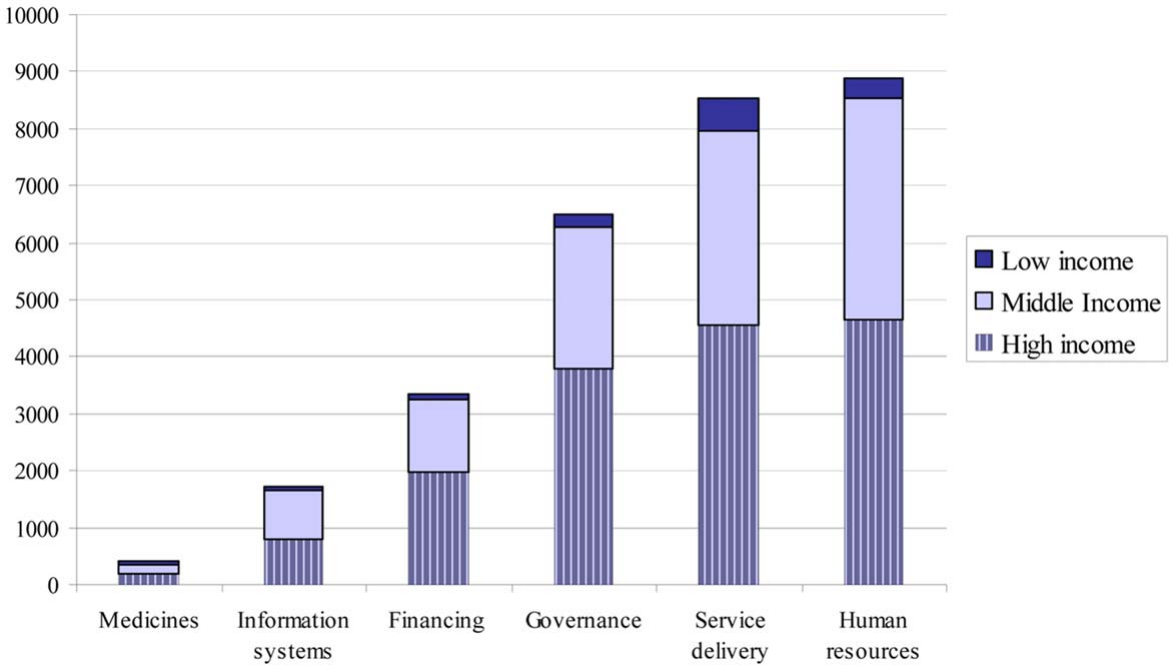
Number of HPSR publications relevant to LMICs by topic and residence of lead author, grouped by income group (2003–2009).

Figure 2 shows the total number of HPSR publications on LMICs by topic, classified by residence of lead author. The total publications between 2003 and 2009 ranged from a low of 648 on medicines to a high of 10357 for service delivery. Taken together, they represent 10% of global publications on these topics, i.e., the vast majority of current HPSR evidence is relevant to HICs, see Table 2. Publications by lead authors from LICs represented only 4% of all HPSR publications in LMICs, ranging from 3% on health financing to 7% on medicines and service delivery, Table 2.

**Table 2:** Number and percent of HPSR publications focusing on Low-income and middle-income country by topic area (2003–2009).

Topic	Publications on HPSR			Percent of publications on LMIC (column b)		
				by residence of Lead author		
	Global (a)	LMIC (b)	%	LIC	MIC	HIC
Human resources	81086	9865	12	4%	42%	55%
Health financing	57173	4638	8	3%	37%	59%
Service delivery	74545	10357	14	7%	40%	53%
Medicines	6280	648	10	7%	43%	51%
Information systems	23164	1877	8	4%	49%	47%
Governance	122587	7911	6	4%	39%	57%
Weighted average across all categories			10	4%	38%	52%

**Figure 3:**
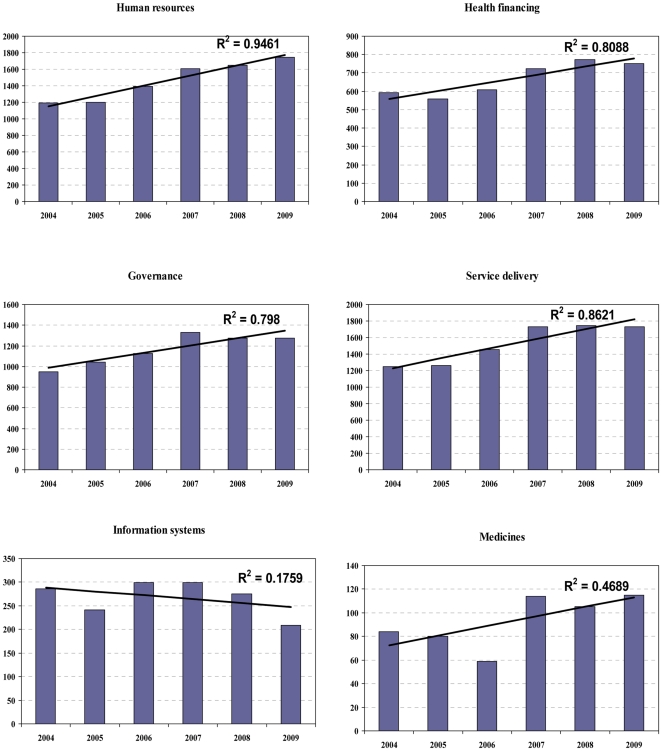
Trends in HPSR publications relevant to LMICs over time by topic area (2003–2009).

**Figure 4:**
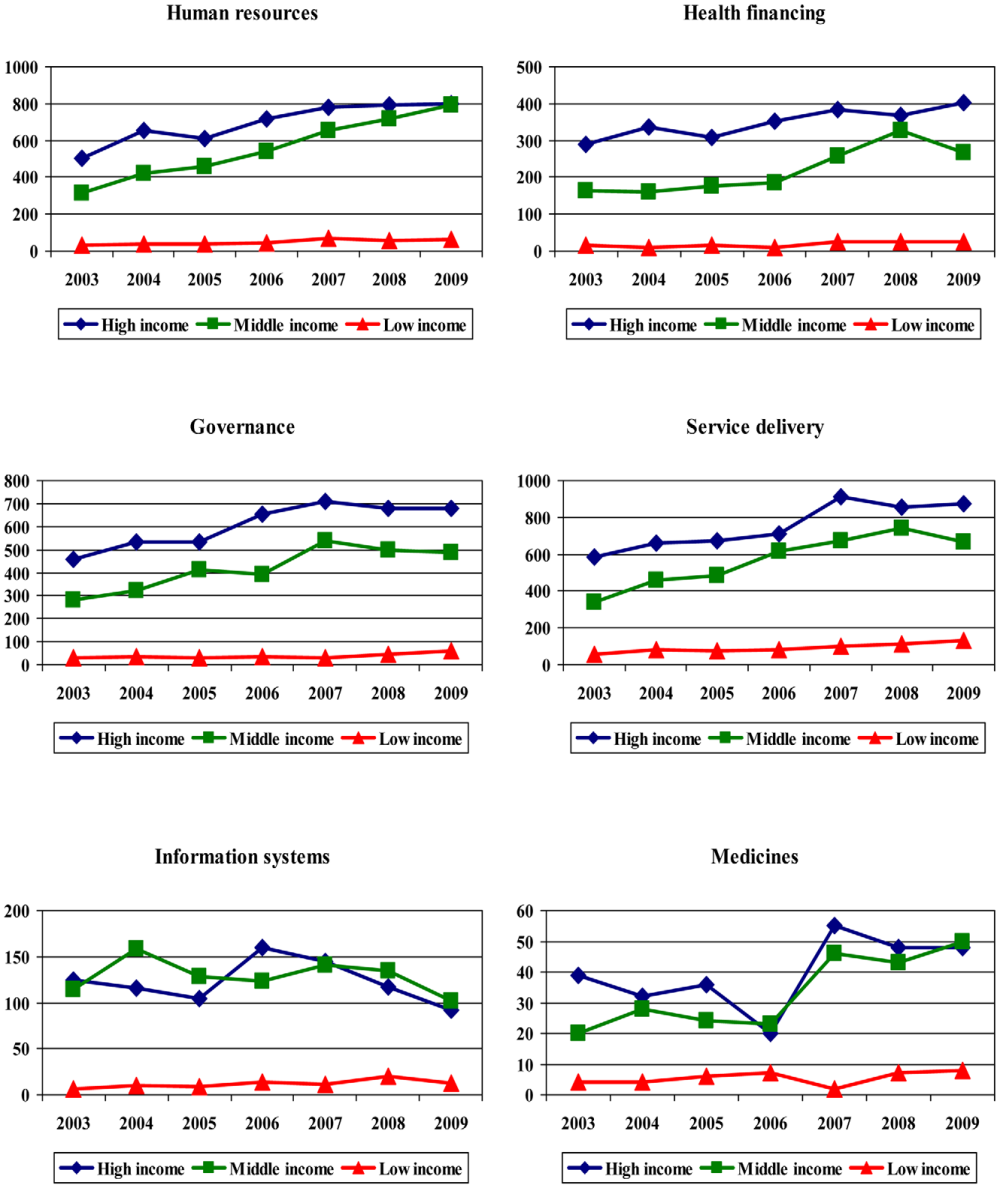
Growth in HPSR publications relevant to LMICs by topic and residence of lead author (2003–2009).

Looking at growth in publications over time, there was an increasing trend in publications focusing on LMIC countries in the six topic areas, except for research on health information systems (Figure 3). However, this was mainly driven by articles whose lead authors were from HIC and MICs, see Figure 4.

The number of HPSR publications focussing on LMICs increased at a faster rate than publications on HPSR in total. For example, in 2009 the number of publications on human resources with a LMIC focus was 2.1 times the number published in 2003, compared with a 1.7 times increase between 2003 and 2009 in non-LMIC contexts. Overall, publications on the 6 topic areas increased by an average of 3% over time between 2003 and 2009.

The bibliometric analysis also provided useful information on the research areas of interest as well as research gaps, an important input into priority setting for research (see Table 3). For example, research on human resources for health focused on training (60%) and migration (32%), while research on distribution and retention of human resources, where health systems in LMICs are still struggling, is notably lagging behind (8%). In service delivery, research on quality of care and performance was the main topic of interest (80%), while access to health services (12%) and the role of non-state sector (8%) were relatively neglected. Research on strengthening the governance and leadership roles of the health system, particularly stewardship roles through licensing and accreditation (2%) and auditing (8%), were also considerably neglected compared with studies on health professional's roles and authority, including the scope, content and location of practice (60%). Finally, research on medicines focused on selection of medicines, e.g., development of essential drug lists or formularies (24%), monitoring, e.g., of adverse drug effects and pharmacovigilence (22%), and medicines regulation and quality assurance (15%), while not much was published on prescribing practices and medicines use (3%), or on marketing (1%) and patient information (1%).

**Table 3:** Distribution of HPSR publications in LMICs by sub-topic (2003–2009)^1^.

Topic	N	%	Topic	N	%
**Human resources**			**Medicines**		
Training	6608	60%	Selection	183	24%
Migration	3561	32%	Monitoring	167	22%
Distribution and retention	846	8%	Regulation and Quality Assurance	115	15%
**Health financing**			Intellectual Property	94	12%
Payment mechanisms	2805	48%	Access	61	8%
Resource allocation	1971	33%	Insurance and Financing	47	6%
Health insurance	1121	19%	Policy/Reform	27	4%
**Governance**			Prescribing and Utilization	26	3%
Professional authority and roles (scope, content and location of practice)	5091	60%	Medicine Supply	26	3%
Government regulation and legislation	1790	21%	Information	9	1%
Consumer involvement	811	10%	Marketing	4	1%
Audit	646	8%	**Service delivery**		
Licensing and accreditation	137	2%	Quality of care and performance	9972	80%
			Access, integrated care, continuum of care and modes of delivery	1438	12%
			Role of the non-state sector	1051	8%

1 the total number of publications in this analysis is higher than the numbers presented in Table 2 as we allowed for multiple categorization of main focus while in Table 2 we ensured that publications were only counted once.

### Institutional survey

Table 4 summarizes information on sources and funding availability for HPSR, as well as collaboration on funded projects between different stakeholders. The total number of observations is higher than the total number of institutions included in the analysis since respondents were asked to provide information on the five most recent projects on HPSR undertaken by their institution during the previous year. In 2010 prices, the mean grant size in LICs doubled and the median increased 10 folds between 2008 and 2010. No major changes were observed in MICs over time, while funding in HICs decreased in the most recent survey compared to 2008. These findings should be interpreted with caution particularly for HICs, where only 7 out of 16 institutions (44%) provided information on this question, which may suggest under reporting. Similarly, data from LICs were available from 6 out of 16 institutions (37%); all except one were in Africa. This is most likely an indication of lack of funded projects during the requested period rather than under-reporting as most of these institutions were either small institutions or ones that were only recently involved in HPSR.

**Table 4:** Research grant funding for HPSR in current and 2010 US$^1^.

	2000			2008			2010	
	LIC	MIC	LIC	MIC	HIC	LIC	MIC	HIC
Number^2^	n = 90	n = 210	n = 41	n = 56	n = 24	n = 16	n = 94	n = 28
**Average grant size and number of grants**	US$	US$	US$	US$	US$	US$	US$	US$
Mean grant size (Current US$)	227,337	100,928	152,598	152,151	1,814,248	397,756	137,135	763,210
Median grant size (Current US$)	34,906	25,555	23,500	30,000	675,000	231,875	50000	250,000
Mean grant size (2010 US$)^1^	284,226	126,184	154,897	154,444	1,841,586	397,756	137,135	763,210
Median grant size (2010 US$)^1^	43,641	31,950	23,854	30,452	685,171	231,875	50,000	250,000
**Source of funding for research grant** 3	%	%	%	%	%	%	%	%
International or bilateral	68	44	78	43	38	88	66	43
National government	11	34	15	43	63	12	22	39
Private	14	1	7	11	8	6	9	18
Other	7	21	5	9	8	0	7	18
**Collaborators on HPSR research** 4	%	%	%	%	%	%	%	%
HIC	13	23	20	20	34	53	31	64
LMIC	36	32	41	38	83	47	23	75
National government	41	51	29	47	34	65	27	43
None	26	21	22	24	0	24	31	0

1 Amounts in 2000 and 2008 US dollars were converted to 2010 US$ using GDP deflators to account for inflation.

2 Number of observations represents HPSR funded projects not the number of institutions included in the survey.

3 Percentages sum to more than 100% as some projects are funded from multiple sources.

4 Percentages sum to more than 100% as some projects have collaborations with more than one entity.

In terms of funding sources, LICs are still mainly funded by international and bilateral organizations (88% in 2010) while government funding is stagnant at around 11%–15%. As in previous years, multiple sources of funding for projects is a prevalent practice in HICs but virtually non-existent in LICs.

With respect to collaborations between HIC and LMIC institutions and national governments, 93% of HIC projects were in collaboration with at least one or more institutions in LMIC, including the national government (Table 4). This was less so in MIC and LIC institutions were around 25% to 30% were not undertaken in collaboration with others. A notable difference between the 2008 and 2010 surveys is a doubling of projects in LIC undertaken in collaboration with the government (65% in 2010 compared with 29% in 2008). Similarly, collaborations between HIC and MIC institutions almost tripled (53% in 2010 compared with 19.5% in 2008).

**Figure 5:**
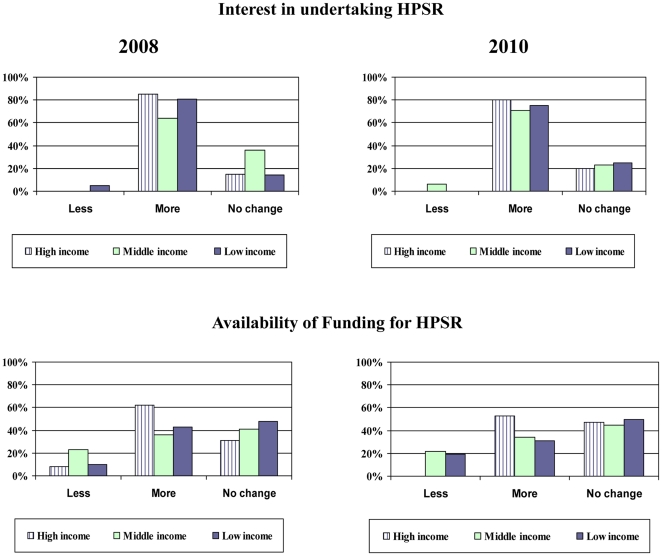
Perception of availability of funding and interest in HPSR in 2008 and 2010.

These findings are consistent with respondents' perceptions of funding availability and interest to undertake HPSR in 2008 and 2010. While their perception of the interest to undertake HPSR remained high in 2010, their perception of funding availability was reduced in 2010 compared with 2008, particularly for LICs, see Figure 5.

Finally, despite an improvement in the availability of infrastructure for research and access to resources in LICs, there is no change in the level of human resource capacity between 2008 and 2010 in all three indicators, namely, percent of directors with more than 10 years experience, percent of staff with PhD and mean number of professional staff involved in HPSR. Access to peer-reviewed publications is still unacceptably low in LICs (62%), but computer and internet access has improved, Table 5.

**Table 5:** Research Capacity in institutions undertaking HPSR.

	2000			2008			2010	
	LIC	MIC	LIC	MIC	HIC	LIC	MIC	HIC
Number	n = 42	n = 69	n = 17	n = 21	n = 11	n = 16	n = 64	n = 16
**Staffing**								
Director has more than 10 years experience (%)	36	52	67	76	92	63	51	87
Staff with PhD (%)	26	24	36	22	66	24	36	58
SD			29	18	21	23	30	31
Mean number of total professional staff (number)	7	13	13	12	36	14	18	21
SD			9	9	27	11	19	20
**Access to resources**								
All researchers have exclusive access to a computer (% institutions)	64	71	67	95	100	94	94	100
All computers linked to internet (% institutions)	31	78	67	95	100	81	95	100
Access to peer reviewed HPSR journals (% institutions)			67	81	100	63	89	100

SD: Standard deviation.

## Discussion

This paper assessed the progress in the field of HPSR over the past decade with respect to three dimensions: trends in peer-reviewed publications on HPSR relevant to LMICs; capacity to undertake HPSR and; funding availability for HPSR. Despite the limitations in this analysis, it provides the most comprehensive and up-to-date assessment of the development of the field of HPSR over the past decade. Among the most important limitations are the low response rate of the 2008 and 2010 institutional surveys, particularly for LICs and HICs, and the limited set of indicators to assess capacity to undertake HPSR. The low response rate is likely due to the sometimes difficult or time-consuming task of collecting institutional-level data on funding for, and capacity to do, HPSR, where non-respondents may not have had the incentive to pursue.

The bibliometric analysis was limited to PubMed and to peer-reviewed publications. In addition, some of the topics may not have been fully represented. For example, publications surrounding trade and pricing relevant to medicines may be under-represented in PubMed as opposed to other search databases. Nonetheless, PubMed is one of the largest search engines hosting more than 5500 journals from over 80 countries and is subscription free which makes it also the most accessible search engine for publications on HPSR in Low-income and middle-income countries.

The bibliometric analysis showed that while peer-reviewed publications on HPSR increased globally over time, the vast majority is relevant to HICs with only 10% of the evidence from LMICs (Table 2). Out of the HPSR publications focusing on LMICs, only 4% were led by researchers from LICs (Table 2). This demonstrates that the concept of the 10–90 gap is still pertinent, even in this area of research where local capacity and context is particularly important [28]. It highlights the importance of more concerted efforts to invest in research led by, and building capacity of, LIC researchers, where the knowledge base evidence-informed decision making is most needed.

The bibliometric analysis also provided useful indication of areas of research interest and gaps. For example there is a 16 fold difference in the number of publications on medicines policies compared with service delivery (Table 2), with research on medicines policies contributing only 2% of all HPSR relevant to LMICs in the analysis period. The picture is also similar for research on health information systems, where it represented 5% of all HPSR relevant to LMICs in the same analysis period. This may be in part due to what funders perceive as priorities for research and the reason why analysis like this is important to highlight such huge gaps and discrepancies. Some of the important research gaps highlighted in our analysis are research around access to health services, the distribution and retention of human resources; and several topics around governance issues (Table 3). Negligible interest in research around health information systems has been also noted, see Figure 3, an illustration of the historical neglect of this area of research [29]. These findings may well be a reflection of the lack of suitable and adapted methods to address complex topics around HPSR and of sufficient funding to address important topics that may be costly to investigate. While research around basic quantitative or descriptive analysis has grown, e.g., assessment of training needs, quality of care or the roles and scope of practice of different providers, more complex issues such as the role of the non-state sector or sensitive issues such as audit are addressed in lesser extent through research (Table 3). It is also a reflection of the research priorities set, or perceived, by major funding sources for HPSR. Our analysis aspires to redress this imbalance by influencing future research priorities in areas where knowledge gaps were identified.

It is arguable that the increase in HPSR research on LMICs peaked around 2006 onwards, see Figure 4. Assuming that the publication cycle takes around two years, on average, from the onset of research till the study is published; this may imply that high-level events and reports, such as the ministerial summit in 2004 and the world health resolution in 2005 played an important role in catalyzing more research in certain areas. However, the slower progress in the most recent years suggests that pledges and commitments need to be monitored by trusted high-profile organizations and the results fed back to the stakeholders involved to ensure meaningful and sustained progress in achieving set goals and commitments.

The institutional survey provided interesting insights into the dynamics of capacity to undertake and funding availability for HPSR. Compared to the 2000 survey, the mean grant size in both the 2008 and 2010 surveys were considerably higher in LICs than in MICs. In 2010, the median grant size in LICs was very close to that of HICs. Since around 70% to 80% of these funds come from international and bilateral sources, this suggests that there was a conscious effort to increase funding allocation to LICs in recent years. International sources of funding have also increased in MICs in 2010 compared with 2008 while domestic sources decreased by half. The increase in funding was not as substantial as in LICs, however. Seeking multiple funding sources was a common practice in MICs but almost non-existent in LICs, possibly due to the limited capacity of LIC institutions. Finally, research collaboration between northern and southern institutions has increased in the two most recent surveys, a fruitful outcome of encouraging or requiring research collaboration in recent funding practices. This has not yet translated to a notable increase in research being led by LIC authors, however, see Figure 4.

Building capacity for research in LMICs has been a topic of concern for a long time, as reflected in several WHO resolutions and global agenda for action [1], [4], [7], [10]. While evidence on effective approaches to build capacity in LMICs is relatively weak, some common conclusions can be identified. Most notably, the critical role of mentoring, a strategy that is less commonly used in LMICs, as an integral part of both short-term and long-term training; as well as using multi-faceted approaches, e.g., combining training with small funding to conduct a research study and having access to a mentor or facilitator during that process [30]. On the funding front, reforming the way international funds are channeled to LMICs may indirectly help increasing domestic funds allocated to research. For example, involving and empowering local stakeholders in setting research priorities; allocating a proportion of international funds to local research teams as core funding; and building capacity at organizational level to understand and use research evidence are all measures that are likely to increase demand and funding for research at national level [31].

In summary, while recognition of the contribution of HPSR to stronger health systems has grown, the human capacity to carry out the research has not kept pace, particularly in LICs. This analysis highlights two important areas to focus efforts in the next few years. The first is to take a more active role, both at national and international levels to identify and fund mechanisms to build research capacity in LICs [30]–[35]. The second is to re-new the commitment to the recommendation made by the Commission on Health Research for Development in 1990 to invest at least 2% of national health expenditures in research and research capacity strengthening [4], [36] and to continue to develop and strengthen not only north-south but also south-south collaborations.

## Supporting Information

Annex S1 **Search strategy for bibliometrics analysis.**
(DOC)
