# Supplementary material for: Trends in Health Policy and Systems Research over the Past Decade: Still Too Little Capacity in Low-Income Countries
Source: PLoS One. 2011 Nov 22;6(11):e27263. doi: 10.1371/journal.pone.0027263 (PMC3222652; doi:10.1371/journal.pone.0027263)
Supplement: Annex S1 — Search strategy for bibliometrics analysis. (DOC) [file pone.0027263.s001.doc]

# Annex S1: Search strategy for bibliometrics analysis

**General inclusion criteria**

1. Studies concerning HPSR
2. Relevant to low- and middle-income countries only
3. Published between 01.01.2003-31.12.2009
4. Limited to human subjects
5. No language limit

*Search terms*

| **Medicines** |
| --- |

1. (drug[ti] or drugs[ti] or pharmaceutical[ti] or pharmaceuticals[ti] or medicines[ti] or medicine[ti])
2. (drug[tiab] or drugs[tiab] or pharmaceutical[tiab] or pharmaceuticals[tiab] or medicines[tiab] or medicine[tiab])
3. (pharmaceutical[ti] or pharmaceuticals[ti] or medicines[ti])
4. (pharmaceutical[tiab] or pharmaceuticals[tiab] or medicines[tiab])
5. **Regulation**
6. ("Drug and Narcotic Control/legislation and jurisprudence"[Mesh] OR "Drug Labeling/ethics"[Mesh] OR "Drug Labeling/legislation and jurisprudence"[Mesh])
7. **Monitoring**
8. ("Drug Monitoring/adverse effects"[Mesh] OR "Drug Monitoring/economics"[Mesh] OR "Drug Monitoring/methods"[Mesh] OR "Drug Monitoring/standards"[Mesh] OR "Drug Monitoring/trends"[Mesh] or "Adverse Drug Reaction Reporting Systems/legislation and jurisprudence"[Mesh] OR "Adverse Drug Reaction Reporting Systems/standards"[Mesh])
9. Pharmacovigilance[tiab]
10. (#6) NOT (#7)
11. (#7) OR (#8)
12. **Selection**
13. ("Formularies, Hospital" [Mesh] OR "Pharmacy and Therapeutics Committee/legislation and jurisprudence "[Mesh])
14. "Drugs, Essential"[majr]
15. ("Essential Drugs"[tiab] OR "Essential Medicines"[tiab])
16. (#11) NOT (#12)
17. (#12) OR (#13)
18. **Insurance and Financing**
19. ("Drug Costs/legislation and jurisprudence"[Mesh] OR " Economics, Pharmaceutical/legislation and jurisprudence"[Mesh] OR "Fees, Pharmaceutical/legislation and jurisprudence "[Mesh])
20. "Rate Setting and Review/legislation and jurisprudence"[Mesh] AND (#2)
21. (reference[ti] OR referencing[ti] AND (price[ti] OR prices[ti] OR pricing[ti])) OR (maximum[ti] AND (price[ti] OR prices[ti] OR pricing[ti])) OR (internal[ti] OR external[ti] AND (price[ti] OR prices[ti] OR pricing[ti]))
22. ("insurance, health, reimbursement"[Mesh] OR "Reimbursement Mechanisms"[Mesh] OR "Cost Sharing"[Mesh]) AND (#4)
23. (copay[ti] OR copays[ti] OR copayment[ti] OR "co pay"[ti] OR "co payment"[ti] OR "co payments"[ti] OR "fees"[ti]) AND (#4)
24. (#18) NOT (#19)
25. (#19) OR (#20)
26. **Intellectual Property**
27. "patents as topic"[Mesh] AND (#1)
28. Trade-Related Aspects of Intellectual Property Rights
29. **Medicines marketing policies**
30. "Social Marketing"[Majr] OR "Advertising as Topic/legislation and jurisprudence"[Mesh] OR "Advertising as Topic/methods"[Mesh] AND (#4)
31. **Medicines information**
32. ("Drug Industry/education"[mesh] OR "Drug Information Services/legislation and jurisprudence"[Mesh])
33. **Prescribing and Use**
34. "Drug Prescriptions/legislation and jurisprudence"[Mesh]
35. "Practice Guidelines as Topic/standards"[Majr] AND (#3)
36. "Drugs, Generic "[MAJR] and (substitute[tiab] or substitution[tiab] or substitutions[tiab])
37. "Drug Utilization/legislation and jurisprudence"[Mesh]
38. "rational use"[ti] and (pharmaceutical[tiab] or pharmaceuticals[tiab] or medicines[tiab])
39. **Medicine Reform/Policy**
40. "Health Policy/legislation and jurisprudence"[Mesh] AND (#3)
41. "Pharmaceutical Services/legislation and jurisprudence"[Mesh]
42. **Medicines access**
43. "Health Services Accessibility"[MAJR] AND (#3)
44. **Medicines supply management**
45. "prescription Drugs/supply and distribution"[Mesh] OR "Nonprescription Drugs/supply and distribution"[Mesh] OR ("Drug Storage/methods"[Majr] OR "Drug Storage/standards"[Majr])

***Medicines (All Categories)***

1. (#5) OR (#9) OR (#10) OR (#14) OR (#15) OR (#16) OR (#17) OR (#21) OR (#22) OR (#23) OR (#24) OR (#25) OR (#26) OR (#27) OR (#28) OR (#29) OR (#30) OR (#31) OR (#32) OR (#33) OR (#34)
2. "Drug Abuse"[tiab] OR "Drug Possession"[tiab] OR "narcotic abuse"[tiab] OR "narcotic use"[tiab] OR narcotics[tiab] OR "substance abuse"[tiab] OR poison[tiab] OR poisoning[tiab] OR venom[tiab] OR "substance use"[tiab] OR cocaine[tiab] OR heroin[tiab] OR marijauna[tiab] OR pot[tiab] OR "salvia divinorum"[tiab]
3. (#35) NOT (#36)

| **Health Financing** |
| --- |

**A. Payment mechanisms**

1. "Fee-for-Service Plans"[Mesh] OR "Physician Incentive Plans"[Mesh] OR Employee Incentive Plans[Mesh] OR "Reimbursement Mechanisms"[Mesh] OR "Reimbursement, Incentive"[Mesh] OR "Prepaid Health Plans"[Mesh] OR "Group Practice, Prepaid"[Mesh] OR "Capitation Fee"[Mesh] OR "Salaries and Fringe Benefits"[Mesh] OR "Financing, Organized"[Mesh] OR "Fees and Charges"[Mesh] OR "Fees, Medical"[Mesh]

**B. Health insurance**

1. "Insurance,Health" [Mesh] OR "Insurance Pools" [Mesh] OR "Insurance Coverage" [Mesh]

**C. Resource Allocation**

1. "Resource Allocation" [Mesh] OR "Health Care Rationing" [Mesh] Or "cost-benefit analysis"[Mesh]

***Health Financing (All Categories)***

1. #1 OR #2 OR #3

| **Human Resources for Health (HRH)** |
| --- |

1. "health manpower" [MeSH]
2. “health personnel" [tiab] OR "health care personnel" [tiab] OR "healthcare personnel" [tiab] OR "medical personnel" [tiab] OR "health professional" [tiab] OR "health care professional" [tiab] OR "healthcare professional" [tiab] OR "medical professional" [tiab] OR "health worker" [tiab] OR "health care worker" [tiab] OR "healthcare worker" [tiab] OR "medical worker" [tiab] OR "health workforce" [tiab] OR "health care workforce" [tiab] OR "healthcare workforce" [tiab] OR "medical workforce" [tiab] or “human resource” [tiab]
3. (#2) NOT (#1)
4. (#1) OR (#3)

# Distribution

1. "Health Services Needs and Demand"[Mesh] OR "Health Care Rationing"[Majr] OR "Resource Allocation"[Majr] OR "Personnel Management"[Majr] OR "Personnel Administration, Hospital"[Majr] OR "Health Resources"[Majr] OR "Job Satisfaction" [Mesh] OR "Burnout, Professional" [Mesh] OR "Personnel Turnover" [Mesh] OR Personnel Staffing and Scheduling[Mesh] OR Career Mobility[Mesh] OR "Personnel Selection"[Mesh]
2. supply[tiab] OR shortage[tiab] OR capacity[tiab] OR employment[tiab] OR distribution[tiab] OR maldistribution[tiab] OR maldistributions[tiab] OR recruit[tiab] OR recruite[tiab] OR recruitement[tiab] OR recruiting[tiab] OR allocation[tiab] OR reallocation[tiab] OR mobility[tiab] OR practice[tiab] OR rural[tiab] OR remote[tiab] OR underserved[tiab] OR (imbalance[tiab] OR retention[tiab] OR "retention strategy"[tiab] OR "financial incentive"[tiab] OR "monetary incentive"[tiab] OR allowances[tiab] OR benefits[tiab] OR "compulsory service"[tiab] OR "bonding scheme"[tiab] OR "vacancy rates"[tiab] OR motivation[tiab]
3. #6 NOT #5
4. #5 OR #7
5. #4 AND #8

# Migration

1. “EMIGRATION AND IMMIGRATION”[MeSH Major Topic] OR "FOREIGN PROFESSIONAL PERSONNEL" [MeSH Major Topic] OR "INTERNATIONAL EDUCATIONAL EXCHANGE" [MeSH Major Topic] OR FOREIGN MEDICAL GRADUATES [MeSH Major Topic]
2. emigration [tiab] OR immigration [tiab] OR migration [tiab] OR "brain drain" [tiab] OR "border crossing" [tiab]
3. #11 NOT #10
4. #10 OR #12
5. **Education I (undergraduate, graduate training**
6. "students"[MeSH Terms] OR "Students, Public Health"[Mesh] OR "Students, Health Occupations"[Mesh] OR "Students, Premedical "[Mesh] OR "Student Dropouts"[Mesh] OR "Health Personnel"[Mesh]
7. "female doctor"[tiab] OR "male nurse"[tiab]
8. #15 NOT #14
9. #14 OR #16
10. “Fellowships and Scholarships” [Mesh] OR "Training Support" [Mesh] OR "Health Planning Support " [Mesh] OR "Financing, Government" [Mesh] OR "Financing, Organized" [Mesh] OR "Financial Support" [Mesh] OR “Personnel Selection” [Mesh] OR Education [Mesh] OR "Education, Distance" [Mesh] OR "Education, Medical" [Mesh] OR "Education, Medical, Undergraduate" [Mesh] OR "Education, Nursing" [Mesh] OR "Education, Nursing, Baccalaureate" [Mesh] OR "Education, Nursing, Associate" [Mesh] OR "Education, Nursing, Diploma Programs " [Mesh] OR "Education, Pharmacy" [Mesh] OR "Education, Dental" [Mesh] OR "Education, Professional" [Mesh] OR Curriculum [Mesh]
11. #17 AND #18
12. **Education II-(pre-service)**
13. "pre service" [tiab] or preservice [tiab] Or "Inservice Training"[Mesh] OR "continuing education"[tiab]
14. #4 AND #20

***Human Resources for Health (All Categories)***

1. (#9 NOT #13) AND (#9 NOT #19) AND (#9 NOT #21)
2. (#13 NOT #9) AND (#13 NOT #19) AND (#13 NOT #21)
3. (#19 NOT #9) AND (#19 NOT #13) AND (#19 NOT #21)
4. (#21 NOT #9) AND (#21 NOT #13) AND (#21 NOT #19)
5. #22 OR #23 OR #24 OR #25

| **Service Delivery** |
| --- |

1. **Access, integrated care, continuity of care, modes of delivery**

1. "Delivery of Health Care"[Mesh:noexp] OR "Delivery of Health Care, Integrated"[Mesh:noexp] OR "Referral and Consultation"[Mesh:noexp] OR "After-Hours Care"[Mesh] OR "Health Care Reform"[Mesh:noexp] OR "Health Services Needs and Demand"[Mesh] OR ("Health Services Accessibility"[Mesh] AND ("Rural Health Services"[Mesh] OR "Adolescent Health Services"[Mesh] OR "Community Health Services"[Mesh] OR "Women's Health Services"[Mesh] OR "ambulatory care"[MeSH Terms:noexp] OR "Home Care Services"[Mesh] OR "Health Facilities"[Mesh] OR "Family Practice"[Mesh] OR "Nursing Services"[Mesh])) OR "Continuity of Patient Care"[Mesh] OR "telemedicine"[MeSH Terms] OR "telemetry"[MeSH Terms] OR ("Telephone"[Mesh] AND "Delivery of Health Care"[Mesh])
2. **Role of non-sector sector**
3. "Private practice" [MeSH] OR " physicians, family "[MeSH] OR " professional corporations "[MeSH] OR " organizations, nonprofit"[MeSH] OR "Outsourced services" [MeSH] OR "Social marketing" [MeSH]
4. "private sector"[tiab] OR "private practitioner"[tiab] OR Private practice[tiab] OR "private provider"[tiab] OR "private providers"[tiab] OR "private provision"[tiab] OR "Non state sector"[tiab] OR "Non state"[tiab] OR "non formal"[tiab] OR informal [tiab] OR traditional[tiab] OR licensed[tiab] OR "non licensed" [tiab]OR unlicensed [tiab] OR "drug vendors"[tiab] OR "medicine sellers"[tiab] OR pharmacists[tiab] OR "NGO"[tiab] OR "Public Private Partnership"[tiab] OR Franchising [tiab] OR Franchizing [tiab] OR Contract*[tiab] OR Contracts [tiab] OR Contracting [tiab]
5. #2 Not #3

# Quality of care and performance

1. "health manpower" [MeSH]
2. “health personnel" [tiab] OR "health care personnel" [tiab] OR "healthcare personnel" [tiab] OR "medical personnel" [tiab] OR "health professional" [tiab] OR "health care professional*" [tiab] OR "healthcare professional*" [tiab] OR "medical professional*" [tiab] OR "health worker*" [tiab] OR "health care worker*" [tiab] OR "healthcare worker*" [tiab] OR "medical worker*" [tiab] OR "health workforce" [tiab] OR "health care workforce" [tiab] OR "healthcare workforce" [tiab] OR "medical workforce" [tiab] or “human resource” [tiab]
3. #6 NOT #5
4. #5 OR #7
5. "Quality Assurance, Health Care"[Mesh] OR "Quality Indicators, Health Care"[Mesh] OR "Quality of Health Care"[Mesh] OR "Health Care Quality, Access, and Evaluation"[Mesh] OR "Quality Control" [Mesh] OR "Total Quality Management" [Mesh] OR "Health Care Evaluation Mechanisms"[Mesh]
6. #8 AND #9

***Service Delivery (All Categories)***

1. (#1 NOT #4) AND (#1 NOT #10)
2. (#4 NOT #1) AND (#4 NOT #10)
3. (#10 NOT #1) AND (#10 NOT #4)
4. (#11) OR (#12) OR (#13)

| **Health Information systems** |
| --- |

1. "Medical Records"[Mesh:NoExp] OR "Medical Records Systems, Computerized" [Mesh] OR "Medical Records, Problem-oriented" [Mesh] OR "Management Information Systems" [Mesh] OR "Drug Information Services" [Mesh]

| **Governance Arrangements** |
| --- |

1. **Government regulation and legislation**
2. "Government Regulation"[Mesh] OR “Legislation as Topic”[Mesh] OR "Legislation, Medical" [Mesh] OR "Legislation, Drug" [Mesh] OR "Legislation, Hospital" [Mesh] OR "Legislation, Pharmacy" [Mesh] OR "Legislation, Dental" [Mesh] OR "Legislation, Nursing" [Mesh] OR "Facility Regulation and Control" [Mesh] OR "Liability, Legal" [Mesh]
3. **Licensing and accreditation**
4. (“Health personnel”[Mesh] AND (Licensure [Mesh] OR Credentialing [Mesh] OR Accreditation [Mesh] ))
5. **Professional authority and roles (scope, content and location of practice)**
6. “Organizational Affiliation" [Mesh:NoExp] OR “Staff Development”[Mesh] OR "Professional Role"[Mesh] OR "Practice Guidelines as Topic"[Mesh] OR "Professional-Patient Relations" [Mesh] OR "Dentist-Patient Relations" [Mesh] OR "Nurse-Patient Relations" [Mesh] OR "Physician-Patient Relations" [Mesh]
7. **Audit**
8. "Management Audit"[Mesh:NoExp] OR "Clinical Audit" [Mesh]
9. **Consumer involvement**
10. "Consumer Participation"[Mesh]

***Governance (All Categories)***

1. (#1 NOT #2) AND (#1 NOT #3) AND (#1 NOT #4) AND (#1 NOT #5)
2. (#2 NOT #1) AND (#2 NOT #3) AND (#2 NOT #4) AND (#2 NOT #5)
3. (#3 NOT #1) AND (#3 NOT #2) AND (#3 NOT #4) AND (#3 NOT #5)
4. (#4 NOT #1) AND (#4 NOT #2) AND (#4 NOT #3) AND (#4 NOT #5)
5. (#5 NOT #1) AND (#5 NOT #2) AND (#5 NOT #3) AND (#5 NOT #4)
6. #6 OR #7 OR #8 OR #9 OR #10

| **Limit to Low- and Middle-Income Countries** |
| --- |

1. "Developing Countries"[Mesh] OR Africa[Mesh] or "Africa South of the Sahara"[Mesh] or Asia[Mesh] or "South America"[Mesh] or "Central America"[Mesh] OR Africa[tiab] or Asia[tiab] or "South America"[tiab] or "Latin America"[tiab] or "Central America"[tiab]
2. "American Samoa"[tiab] or Argentina[tiab] or Belize[tiab] or Botswana[tiab] or Brazil[tiab] or Bulgaria[tiab] or Chile[tiab] or Comoros[tiab] or Costa Rica[tiab] or Croatia[tiab] or Dominica[tiab] or Equatorial Guinea[tiab] or Gabon[tiab] or Grenada[tiab] or Hungary[tiab] or Kazakhstan[tiab] or Latvia[tiab] or Lebanon[tiab] or Libya[tiab] or Libia[tiab] or Libyan[tiab] or Lithuania[tiab] or Malaysia[tiab] or Mauritius[tiab] or Mexico[tiab] or Micronesia[tiab] or Montenegro[tiab] or Oman[tiab] or Palau[tiab] or Panama[tiab] or Poland[tiab] or Romania[tiab] or Russia[tiab] or Seychelles[tiab] or Slovakia[tiab] or South Africa[tiab] or "Saint Kitts and Nevis"[tiab] or "Saint Lucia"[tiab] or "Saint Vincent and the Grenadines"[tiab] or Turkey[tiab] or Uruguay[tiab] or Venezuela[tiab] or Yugoslavia[tiab] or Mayotte[tiab] or "Northern Mariana Islands"[tiab] or "Russian Federation"[tiab] or Samoa[tiab] or Serbia[tiab] or "Slovak Republic"[tiab] or "St Kitts and Nevis"[tiab] or "St Lucia"[tiab] or "St Vincent and the Grenadines"[tiab]
3. Albania[tiab] or Algeria[tiab] or Angola[tiab] or Armenia[tiab] or Azerbaijan[tiab] or Belarus[tiab] or Bhutan[tiab] or Bolivia[tiab] or "Bosnia and Herzegovina"[tiab] or Bosnia[tiab] or Cameroon[tiab] or China[tiab] or Colombia[tiab] or Congo[tiab] or Cuba[tiab] or Djibouti[tiab] or "Dominican Republic"[tiab] or Ecuador[tiab] or Egypt[tiab] or El Salvador[tiab] or Fiji[tiab] or "Georgia (Republic)" [tiab] or Guam[tiab] or Guatemala[tiab] or Guyana[tiab] or Honduras[tiab] or "Indian Ocean Islands"[tiab] or Indonesia[tiab] or Iran[tiab] or Iraq[tiab] or Jamaica[tiab] or Jordan[tiab] or Lesotho[tiab] or "Macedonia" [tiab] or "Marshall Islands"[tiab] or Micronesia[tiab] or "Middle East"[tiab] or Moldova[tiab] or Morocco[tiab] or Namibia[tiab] or Nicaragua[tiab] or Paraguay[tiab] or Peru[tiab] or Philippines[tiab] or Samoa[tiab] or "Sri Lanka"[tiab] or Suriname[tiab] or Swaziland[tiab] or Syria[tiab] or Thailand[tiab] or Tonga[tiab] or Tunisia[tiab] or Turkmenistan[tiab] or Ukraine[tiab] or Vanuatu[tiab] or "Cape Verde"[tiab] or Gaza[tiab] or Georgia[tiab] or Kiribati[tiab] or Macedonia[tiab] or Maldives[tiab] or Palestine[tiab] or "Syrian Arab Republic"[tiab] or "West Bank"[tiab]
4. Afghanistan[tiab] or Bangladesh[tiab] or Benin[tiab] or "Burkina Faso"[tiab] or Burundi[tiab] or Cambodia[tiab] or "Central African Republic"[tiab] or Chad[tiab] or Comoros[tiab] or "Democratic Republic of the Congo"[tiab] or "Cote d'Ivoire"[tiab] or Eritrea[tiab] or Ethiopia[tiab] or Gambia[tiab] or Ghana[tiab] or Guinea[tiab] or Guinea-Bissau[tiab] or Haiti[tiab] or India[tiab] or Kenya[tiab] or Korea[tiab] or Kyrgyzstan[tiab] or Laos[tiab] or Liberia[tiab] or Madagascar[tiab] or Malawi[tiab] or Mali[tiab] or Mauritania[tiab] or Melanesia[tiab] or Mongolia[tiab] or Mozambique[tiab] or Myanmar[tiab] or Nepal[tiab] or Niger[tiab] or Nigeria[tiab] or Pakistan[tiab] or "Papua New Guinea"[tiab] or Rwanda[tiab] or Senegal[tiab] or "Sierra Leone"[tiab] or Somalia[tiab] or Sudan[tiab] or Tajikistan[tiab] or Tanzania[tiab] or East Timor[tiab] or Togo[tiab] or Uganda[tiab] or Uzbekistan[tiab] or Vietnam[tiab] or Yemen[tiab] or Zambia[tiab] or Zimbabwe[tiab] or Burma[tiab] or Congo[tiab] or Kyrgyz[tiab] or Lao[tiab] or "North Korea"[tiab] or "Solomon Islands"[tiab] or "Sao Tome"[tiab] or Timor[tiab] or "Viet Nam"[tiab]
5. "developing country"[tiab] OR "developing countries"[tiab] OR "developing nation*"[tiab] OR "less* developed country"[tiab] OR "less* developed countries"[tiab] OR "under developed country"[tiab] OR "under developed countries"[tiab] OR "poor* country"[tiab] OR "poor* countries"[tiab]
6. "middle income country"[tiab] or "middle income countries"[tiab] or "low income country"[tiab] or "low income countries"[tiab]
7. lmic[tiab] or lmics[tiab]
8. (#1) OR (#2) OR (#3) OR (#4) OR (#5) OR (#6) OR (#7)

-------------------------------------------------------------------------------------------------

*Additional steps for "Medicines" only*

1. Japan[tiab] OR "United States"[tiab]
2. **(**#8) NOT (#9)

| **Limit Publication Dates and to "Humans"** |
| --- |

**Publication Date : 01/01/2003 to 31/12/2009**

1. Animals[mh]
2. Humans[mh]
3. #1 not (#1 and #2)
4. (#2) NOT (#3)
